# Child/Adolescent Anxiety Multimodal Study (CAMS): rationale, design, and methods

**DOI:** 10.1186/1753-2000-4-1

**Published:** 2010-01-05

**Authors:** Scott N Compton, John T Walkup, Anne Marie Albano, John C Piacentini, Boris Birmaher, Joel T Sherrill, Golda S Ginsburg, Moira A Rynn, James T McCracken, Bruce D Waslick, Satish Iyengar, Phillip C Kendall, John S March

**Affiliations:** 1 Duke University Medical Center, Department of Psychiatry and Behavioral Sciences, DUMC Box 3527, Durham, NC 27710, USA; 2 The Johns Hopkins Hospital, Division of Child and Adolescent Psychiatry, 600 North Wolfe Street, Baltimore, MD 21287, USA; 3 New York State Psychiatric Institute-Columbia University Medical Center, 1051 Riverside Drive, New York, NY 10032, USA; 4 University of California at Los Angeles, Semel Institute for Neuroscience and Human Behavior, 760 Westwood Plaza, 68-251B, Los Angeles, CA 90095, USA; 5 Western Psychiatric Institute and Clinic-University of Pittsburgh Medical Center, 3811 O'Hara Street, Pittsburgh, PA 15213, USA; 6 Division of Services and Intervention Research, National Institute of Mental Health, 6001 Executive Boulevard, MSC 9633, Bethesda, MD 20892, USA; 7 Baystate Medical Center, 759 Chestnut Street, Springfield, MA 01199, USA; 8 Temple University, Department of Psychology, Weiss Hall 1701 North 13th Street, Philadelphia, PA 19122, USA

## Abstract

**Objective:**

To present the design, methods, and rationale of the Child/Adolescent Anxiety Multimodal Study (CAMS), a recently completed federally-funded, multi-site, randomized placebo-controlled trial that examined the relative efficacy of cognitive-behavior therapy (CBT), sertraline (SRT), and their combination (COMB) against pill placebo (PBO) for the treatment of separation anxiety disorder (SAD), generalized anxiety disorder (GAD) and social phobia (SoP) in children and adolescents.

**Methods:**

Following a brief review of the acute outcomes of the CAMS trial, as well as the psychosocial and pharmacologic treatment literature for pediatric anxiety disorders, the design and methods of the CAMS trial are described.

**Results:**

CAMS was a six-year, six-site, randomized controlled trial. Four hundred eighty-eight (N = 488) children and adolescents (ages 7-17 years) with DSM-IV-TR diagnoses of SAD, GAD, or SoP were randomly assigned to one of four treatment conditions: CBT, SRT, COMB, or PBO. Assessments of anxiety symptoms, safety, and functional outcomes, as well as putative mediators and moderators of treatment response were completed in a multi-measure, multi-informant fashion. Manual-based therapies, trained clinicians and independent evaluators were used to ensure treatment and assessment fidelity. A multi-layered administrative structure with representation from all sites facilitated cross-site coordination of the entire trial, study protocols and quality assurance.

**Conclusions:**

CAMS offers a model for clinical trials methods applicable to psychosocial and psychopharmacological comparative treatment trials by using state-of-the-art methods and rigorous cross-site quality controls. CAMS also provided a large-scale examination of the relative and combined efficacy and safety of the best evidenced-based psychosocial (CBT) and pharmacologic (SSRI) treatments to date for the most commonly occurring pediatric anxiety disorders. Primary and secondary results of CAMS will hold important implications for informing practice-relevant decisions regarding the initial treatment of youth with anxiety disorders.

**Trial registration:**

ClinicalTrials.gov NCT00052078.

## Introduction

The purpose of this manuscript is to describe the research design, rationale for the design choices, and methods used to implement the Child/Adolescent Multimodal Study (CAMS), a recently completed federally-funded, multicenter, randomized comparative treatment trial that examined the short-term efficacy (12-weeks) and long-term durability (36-weeks) of four treatments for childhood and adolescent separation anxiety disorder (SAD), generalized anxiety disorder (GAD), and social phobia (SoP): cognitive-behavioral therapy (CBT), sertraline (SRT), their combination (COMB), and pill placebo (PBO). The methodological challenges faced while developing and implementing the trial are also discussed.

## Study Rationale

Anxiety disorders in children and adolescents are prevalent, [1] impairing, [2] and often precursors to psychiatric disorders in later adolescence and adulthood including additional subsequent anxiety disorders, major depression, substance abuse, and suicide attempts [3,4]. With the exception of specific phobias, SAD, GAD, and SoP are the most common triad of anxiety diagnoses in both community and clinical samples of children and adolescents [5]. Pediatric anxiety disorders are highly comorbid with one another as well as with other psychiatric disorders such as attention-deficit/hyperactivity disorder, major depression, and dysthymia [1,6]. Given their high prevalence and psychiatric comorbidity, anxiety disorders in children and adolescents often results in impairment and distress that significantly interferes with family, academic, and social functioning [1,2,7].

The past two decades witnessed critical scientific advances in the treatment and understanding of anxiety disorders in youth that laid the groundwork for the launch of the CAMS trial. These advances included: (1) a better understanding of the public health importance of anxiety disorders in children and adolescents; [8,9] (2) the development of valid and reliable anxiety specific multi-informant and multi-method assessments (without which research in pediatric anxiety would not be possible); (3) a growing empirical literature base supporting the short-term efficacy and feasibility of both psychosocial [10] and psychopharmacological[11] interventions for the treatment of anxiety disorders in youth; (4) room for improved outcomes in monotherapies[12] suggesting that current treatments are prime candidates for innovation;[13] (5) paucity of studies comparing the efficacy of combination treatment (e.g., cognitive-behavioral therapy plus medication) using a credible control condition in the same patient population; [13] and (6) general agreement within the scientific community that the results of a large comparative treatment trial like CAMS could meaningfully impact public policy [14].

With these factors in mind, the National Institutes of Mental Health (NIMH) funded the CAMS trial to further scientific knowledge on effective treatments for pediatric anxiety disorders. CAMS was a 6-year, multisite (6 sites), randomized controlled trial (RCT). Four hundred and eighty-eight children and adolescents between the ages of 7-17 years with at least one DSM-IV-TR diagnoses of SAD, GAD, or SoP were randomly assigned to one of four treatment groups: CBT, SRT, COMB or pill PBO. Results of the primary outcomes were recently published[15] and showed that at the end of 12 weeks of acute treatment 80.7% (95% CI, 73.3 to 86.4%) of participants treated with COMB were rated as treatment responders (defined as a Clinical Global Impression-Improvement (CGI-I) score of 1 or 2) [16]. COMB was superior to both CBT alone (59.7%; 95% CI, 51.4 to 67.5%, p < 0.001) and SRT alone (54.9%; 95% CI, 46.4 to 63.1%, p < 0.001), as well as pill placebo (23.7%, 95% CI, 15.5 to 34.5%, p < 0.0001). CBT alone and SRT alone were also superior to pill placebo (p < 0.001, p < 0.001, respectively) but not statistically significantly different from one another (p = 0.41). A similar pattern of response was found on the Pediatric Anxiety Rating Scale (PARS),[17] a clinician administered scalar assessment scale. The overall findings from the acute phase of the CAMS study suggest that there are three effective treatments for youth suffering from one or more of the target anxiety disorders, with COMB being the most effective.

### Rationale for the CAMS Treatments

At the time CAMS was initiated, cognitive-behavior therapy [18-20] and selective serotonin reuptake inhibitors [21-24] had emerged as the most effective treatments for pediatric anxiety disorders [25]. Despite positive outcomes in previous RCTs,[12] response rates were short of exemplary, with approximately 40-50% of treated youth remaining symptomatic at the end of acute treatment. Moreover, with the exception of one small study[26] that compared CBT alone to medication alone in youth with SoP, clinical trialists had not yet compared the relative efficacy of psychosocial and psychopharmacological interventions in the same study population. This had raised speculation that CBT trials (often based in university psychology clinics) and medication trials (often based in medical centers) were conducted with different populations of anxious youth.

With respect to combination trials for childhood anxiety disorders, only one study, conducted in a pediatric obsessive-compulsive disorder (OCD) population,[27] compared and demonstrated the superiority of combination treatment (CBT+SSRI) to CBT and SSRIs alone. Therefore, CAMS provided an important and necessary extension to the empirical literature by comparing CBT alone, an SSRI alone, and their combination to pill placebo in the same clinical population recruited across both medical center and psychology clinic sites.

### Cognitive-Behavioral Therapy Studies

Cognitive behavioral therapy for child and adolescent anxiety disorders assumes that pathological anxiety is the result of an interaction between somatic or physiological arousal, cognitive distortions, and avoidance behavior. Accordingly, CBT [28] addresses each domain through: (1) corrective psychoeducation about anxiety and feared situations; (2) developmentally appropriate cognitive restructuring skills to address maladaptive thinking and to learn coping-focused thinking; (3) somatic management techniques to target autonomic arousal and related physiological reactivity; (4) graduated, systematic, and controlled exposure tasks to feared situations/stimuli to eliminate avoidance behavior; and (5) relapse prevention to consolidate and maintain treatment gains.

To date, over 25 RCTs have evaluated CBT for the treatment of anxiety disorders in youth [13]. The first and most well-studied CBT program for childhood anxiety disorders is Kendall's Coping Cat [18,19]. In two initial trials, children treated with this protocol demonstrated significant improvement on self- and parent-reported measures of distress and coping, as well as clinician ratings of child behavior and diagnostic status when compared to waitlist controls. Benefits have been shown to maintain over long-term follow-up of 7.4 years [29]. Other controlled trials support the efficacy of CBT in childhood anxiety for a wide range of ages (7-17), conditions (OCD, SoP, SAD), and formats (group, individual, and family) (see Silverman) [30].

Limitations of some studies in the CBT literature include the use of completer rather than intent-to-treat (ITT) samples, inclusion of participants with mild anxiety or phobias, failure to track comorbid anxiety and mood disorders, and relatively weak control conditions including wait list and potentially active psychoeducation controls [25,31].

### Pharmacotherapy Studies

Pharmacological treatment in children and adolescents is supported by data suggesting the continuity of childhood anxiety disorders with adult anxiety and depressive disorders [32-35] and efficacy of a range of antidepressant medications in the treatment of adult anxiety disorders, including SSRIs [36]. Prior to CAMS, controlled trials of SSRIs in childhood anxiety disorders support the short-term efficacy and safety of these compounds for the disorders targeted in CAMS, [21-24] as well as for selective mutism[33] and OCD [35,37].

Setting the stage for the pharmacological protocol used in CAMS was the Research Units in Pediatric Psychopharmacology (RUPP) Anxiety Group [23]. These investigators conducted a randomized, double-blind comparison of fluvoxamine and pill placebo in children and adolescents between the ages of 7 to 17 with SAD, GAD, and SoP. Results showed fluvoxamine (a SSRI) was significantly more effective than pill placebo in reducing anxiety symptoms (ES = 1.1). However, limitations of the RUPP study included use of clinician rather than independent evaluator ratings of treatment response and, similar to CBT trials, a substantial portion of subjects remained symptomatic following treatment.

Despite these and other studies showing the anxiolytic benefits of SSRIs, concerns with pharmacologic treatments remain, including the lack of information about the long-term safety and durability of medication treatments for children with anxiety disorders. The black box FDA warnings for the use of SSRI medications in children and adolescents[38] coincided with the CAMS trial and underscored the need for careful procedures to study SSRI safety in children participating in CAMS.

### Comparative Treatment Trials

CAMS is the fifth federally-funded, large, multicenter, comparative treatment trial addressing prevalent and disabling mental health conditions in children and adolescents, and joins ranks with the other large comparative treatment trials: Multimodal Treatment of Children with ADHD Study (the MTA),[39] Treatment for Adolescents with Depression Study (TADS),[40] Pediatric OCD Treatment Study (POTS),[27] and Treatment of Resistant Depression in Adolescents (TORDIA) [41]. Each of these large multisite comparative treatment trials evaluated the most promising psychosocial and pharmacological treatments for their time and targeted psychiatric disorder. These landmark clinical trials have had significant public health value by addressing compelling practice-relevant questions (e.g., what treatment should be provided first to a particular child?) and demonstrated the added benefit of combination treatments. Equally important, analyses of secondary outcomes and moderator and mediators of treatment response have, and will continue to, provided clinically-relevant information for matching patient characteristics to treatment modality to better personalize care and maximize patient outcomes.

## Specific Aims and Design

The specific aims of CAMS were:

Aim 1. To compare the relative efficacy of each active treatment (COMB, SRT, CBT) against PBO in reducing anxiety symptoms and associated disability over 12 weeks of acute treatment.

Aim 2. To compare the relative efficacy of each monotherapy (SRT, CBT) against COMB in reducing anxiety symptoms and associated disability over 12 weeks of acute treatment.

Aim 3. To identify predictors, moderators, and potential mediators of acute response to treatment.

Aim 4. To identify differences in rate of response, dropout, premature termination, safety and adverse events, and consumer satisfaction.

Aim 5. To explore the impact of COMB, SRT, and CBT over a 6-month open follow-up period on functioning, relapse and recurrence rates, and utilization of other treatments.

Each of the above aims was addressed through a two-phase clinical trial. Phase I involved a 12-week randomized controlled trial comparing CBT, SRT, COMB, against pill PBO. Phase II was a 6-month treatment maintenance period, in which Phase I treatment responders were seen by their Phase I clinician(s) monthly. Participants assigned to CBT received monthly booster sessions, while those assigned to SRT received monthly medication monitoring visits. Phase I non-responders to active treatments were referred to community providers. However, Phase I PBO non-responders were provided their choice of an active CAMS treatment at the end of Phase I or at any time during Phase I if their symptoms worsened. In as much as possible (e.g., with the exception of study dropouts), all subjects were evaluated at scheduled assessment points (Weeks 0, 4, 8, 12, 24, and 36) regardless of initial treatment response or participation in Phase II CAMS booster sessions or non-CAMS treatments. In addition to parent, child, and clinician questionnaires that evaluated changes across a wide variety of domains, primary outcomes were assessed by blind independent evaluators (IEs).

**Figure 1:**
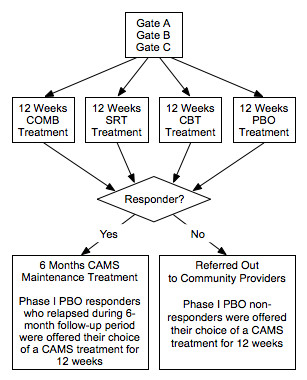
**Child/Adolescent Anxiety Multimodal Study (CAMS) Experimental Design**.

A schematic representation of the study is presented in Figure 1. The six performance sites involved in the trial were: New York State Psychiatric Institute; Duke University Medical Center; Johns Hopkins Medical Institutions; Temple University; University of California at Los Angeles; and Western Psychiatric Institute and Clinic. A multi-layered administrative structure with representation from all sites facilitated cross-site coordination and quality assurance. The CAMS Executive Committee (EC) was comprised of a chair (Dr. John Walkup), co-chair (Dr. Anne Marie Albano), executive secretary (Dr. Scott Compton), lead study coordinator (Dr. Courtney Keeton), and representation from NIMH (Dr. Joel Sherrill). The EC met weekly via teleconference calls and was responsible for overseeing the successful and consistent implementation of the study protocol across all performance sites.

**Figure 2:**
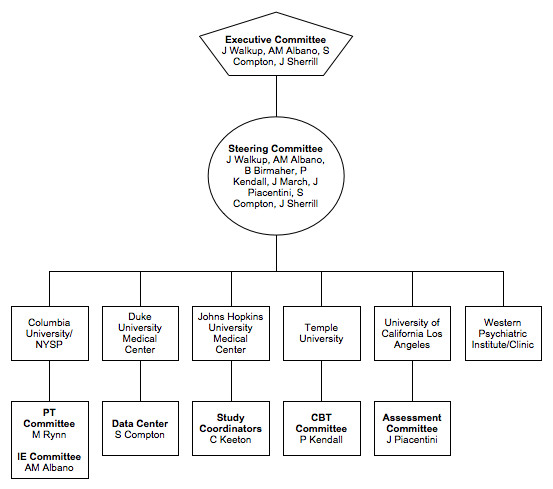
**Child/Adolescent Multimodal Study (CAMS) Organizational Structure and Performance Sites**.

Another essential governing committee, the CAMS Steering Committee (SC), was comprised of principle investigators, co-investigators, and study coordinators from each site. The SC met weekly via teleconference calls to review recruitment progress at each site, discuss and clarify questions sites might have regarding the implementation of the protocol, address clinical concerns with study participants, and report and discuss adverse events and protocol deviations (if any). Subcommittees for each treatment modality were also created. The CBT and PT (pharmacotherapy) committees were comprised of CAMS treatment clinicians, and met separately via teleconference calls to provide cross-site supervision and present and discuss clinical cases on a rotating basis. The CBT committee held weekly conference calls, while the PT committee held bi-weekly conference calls. The difference in the frequency of the calls between the committees was due to differences in the frequency of treatment visits. Participants assigned to CBT met weekly with their therapist, while participants assigned to PT met bi-weekly, with the exception of the first four treatment visits, which were weekly. The IEs also met bi-weekly via teleconference calls for cross-site supervision. The goal of these meetings was to ensure that the assessments were administered similarly across the 6 performance sites. Each performance site also had cross-site responsibilities. Trial wide study coordination was the responsibility of study staff at Johns Hopkins Medical Institutions. CBT, PT, and IE quality assurance was the responsibility of study staff at Temple University, University of California at Los Angeles, and New York State Psychiatric Institute, respectively. Western Psychiatric Institute and Clinic (first three years) and Duke University Medical Center (last three years) served as the data centers (Note: the data center was moved to Duke University Medical Center because of unplanned personnel changes at the original site). A schematic representation of the organizational structure of CAMS and list of performance sites is presented in Figure 2.

### Randomization and Enrollment

To maintain reasonably good balance among the four treatment groups, participants were randomized using a stratified block randomization procedure. Factors utilized in this procedure were treatment site, age, and gender.

Subjects were enrolled using a multiple gating procedure in which parents/guardians of prospective participants first completed an initial telephone screen (Gate A). Following the phone screen, those families who met basic eligibility criteria were then invited to the site's clinic. At the clinic, informed consent and assent were obtained and then the IEs conducted a structured diagnostic interview (Gate B; note: the same IE conducted all future assessments). If the child or adolescent met all inclusion criteria, and no exclusion criteria, a baseline assessment and randomization visit was scheduled a week later (Gate C1 and C2). Immediately following the baseline assessment (Gate C1), the family met with the Principle Investigator (or his/her designate) to answer any remaining questions the family might have about the study. A secondary, yet important, purpose of this meeting was to make certain the family understood how participation in a clinical trial differed from standard clinical care (e.g., treatment followed a standardized research protocol with appropriate clinical safeguards) and to ensure the family was willing to accept randomization to a treatment condition (even if the family had a preference for a particular treatment). Upon completion of this informal re-consenting procedure, participant's randomization was revealed. The first treatment visit typically followed immediately after Gate C2. The expected average time from Gate A to Gate C2 (randomization) was 2-3 weeks, with a range of 2 (minimum) to 6 (maximum) weeks.

Participants completed their 12-week assessment (end of acute treatment) by day 84 (± 5 days). At the end of Phase I, participants in the medication-only treatment conditions (either SRT or PBO) were unblinded; however, the IE remained blinded to treatment status throughout the entire trial. Based upon the IE evaluation of clinical response at the week 12 assessment, responders (defined as CGI-I ≤ 2) entered Phase II. Non-responders (CGI-I > 2) to any of the active treatments were referred to the community for treatment or follow-up care. PBO non-responders (CGI-I > 2) met with their clinician and were offered their choice of a CAMS treatment (e.g., CBT, SRT, or COMB) for an additional 12 weeks. This treatment was provided during Phase II by CAMS study clinicians. PBO responders entered Phase II and continued to meet monthly with their clinician. If at any time during Phase II, PBO responders relapsed, they received the same option of their choice of a CAMS treatment for an additional 12 weeks.

During Phase II, those participants receiving medication (e.g., participants in COMB or SRT) remained at their Week 12 dose of mediation. Downward medication adjustments were allowed in response to emergent side effects. Participants who required a medication dose increase during Phase II were prematurely terminated from the study and continued with the assigned assessments. Participants categorized as CBT responders met with their clinician for monthly 50-minute maintenance CBT sessions. During these sessions no new material was introduced, but the CBT therapist was permitted to revisit the stimulus hierarchy, and reinforce the necessity of exposure activities to promote maintenance and generalization. Responders in the COMB group received both continued stable medication as well as monthly CBT maintenance visits. At the end of Phase II all subjects met with his/her clinician(s) and were given end-of-treatment recommendations and, if necessary, referrals for continued clinical care.

### Design Rationale

As a group, the CAMS investigators had substantial experience with multicenter comparative treatment trials and carefully considered several different treatment designs before deciding upon the final design for CAMS. At the time CAMS was developed, there were realistically three design choices that would allow for a comparison of the two monotherapies and their combination. First, a 1 × 4 parallel groups design (CBT vs. SRT vs. SRT+CBT vs. PBO). This design had been used successfully in several previously funded NIMH trials (e.g., MTA,[39] TADS,[40] and POTS[27]) and had established a precedent for large multicenter comparative treatment trials. Second, a 1 × 5 parallel groups design (CBT vs. SRT vs. SRT+CBT vs. PBO+CBT vs. PBO) that added a balanced pill PBO+CBT condition, for which there was little precedent in the research literature. This design was carefully considered due to concerns about the lack of a PBO+CBT control condition in the first design option. And third, a fully 2 × 2 factorial design (Factor One: CBT vs. Sham CBT; Factor Two: Active Medication vs. Pill PBO). After thorough consideration of the scientific merits and feasibility of implementation of each of these designs, CAMS investigators chose the unbalanced 1 × 4 parallel groups design with pill PBO as the control condition as the best option. The pill PBO condition was deemed necessary to protect against a failed trial and to control for the effect of positive engagement on the part of clinicians and treatment expectancies on the part of participants and parents.

The 1 × 5 parallel groups design was a serious contender but ultimately rejected due to cost and the inherent difficulty of creating a credible and inert sham psychosocial treatment condition [31]. The addition of a PBO+CBT treatment arm would have increased the total cost of the trial by approximately 4 million US dollars. For example, to be able to detect a between group difference of at least 10% between the active treatments would require an increase in sample size from approximately 140 to approximately 290 participants per active treatment group. Thus, the total sample size required to complete the trial would have increased from 480 to 1015 participants, again cost prohibitive. A fully factorial design, although scientifically attractive, was rejected as it was deemed better suited for a true efficacy study given that one of the treatment arms would have been the pill PBO and shame psychosocial treatment. Ultimately, despite its known limitations, [42] CAMS followed in the footsteps of other large pediatric comparative treatment trials and chose an unbalanced 1 × 4 parallel groups design because it represented the best compromise between ecological validity, feasibility of implementation, scientific rigor, and cost.

Other alternative designs choices were also considered for the follow-up maintenance period (Phase II) of the CAMS trial, but ultimately rejected due to feasibility, cost (e.g., re-randomizing Phase I non-responders to new treatments), and ethical considerations (e.g., continuing PBO throughout phase II).

### Decision to Focus on Three Anxiety Disorders

The decision to target children and adolescents with DSM-IV-TR SAD, GAD, or SoP was made for both pragmatic and theoretical reasons. From a pragmatic perspective, there was a strong historical precedent to study these three disorders in the same trial [10,13,23,30]. SAD, GAD, and SoP share a similar response to CBT and SSRI treatments, exhibit strong associations with each other (comorbidity), and as a group, have historically been considered distinct from other childhood-onset DSM-IV anxiety disorders (e.g., OCD or PTSD).

### Primary Outcome Measures

CAMS had two primary outcome measures, one categorical and one continuous: (1) responder status (i.e., "responder" or "non-responder") based on the 7-point Clinical Global Impression-Improvement Scale [16]. (CGI-I) score of 1 ("very much improved") or 2 ("much improved"); and (2) the total score on the Pediatric Anxiety Rating Scale (PARS) [17]. Scores on both outcome measures were based on an interview with the child and parent(s) by the IE. The child and parent(s) were interviewed together as is standard practice for the PARS (Note: whenever possible, ADIS assessments were conducted separately). IEs were trained to handle questions of adolescent confidentiality and parent-child conflict that arose at times during joint interviewing.

The inability to fully mask the CBT and COMB conditions in other pediatric comparative clinical trials has been criticized because of the potential for differential expectancy effects and differences in time and attention provided by clinicians [42]. From a pure efficacy perspective these criticisms are valid. However, in CAMS the goal was ecological validity with an emphasis on effectiveness in as much was feasible. Moreover, masking of the primary outcome variables was maintained by the use of independent evaluators who were blind to treatment status. Thus, the use of blind IEs removed rater expectancy as a source of potential bias in outcomes.

Efforts to ensure that IEs maintained blindness were multifaceted. First, on-site supervision for IEs was held separate from any meetings with treatment clinicians. For example, during each site's weekly research meeting, IEs were excused from the meeting when the focus of the meeting shifted to discussing clinical information about study participants. Second, IE offices were required to be in a location separate from the offices of clinicians and other study staff (e.g., in a different area or floor of the building). Third, all study staff were trained to assist in maintaining the blind and participants were repeatedly reminded to refrain from discussing or mentioning their study treatment. Fourth, given the significant experience CAMS investigators had with prior clinical trials, rigor in maintaining the blind was as good or better than previous multisite trials, although as with any clinical trial there was the occasional error. To assess the impact that unblinding may have had on outcomes, IEs were asked to complete a questionnaire following the week 12 assessment which asked them to guess which treatment the participant received and indicate their degree of confidence in this rating. Given the rigorous efforts to maintain the blind, the frequency of incidents that led to breaking the blind (e.g., seeing the participant with a therapist) was minimal.

Participants were encouraged to complete all scheduled assessments and were compensated for time and travel consistent with local IRB guidelines. Participants who withdrew from treatment at any point during Phase I or Phase II were asked if they would be willing to complete all future assessments, and if so, they were classified as "treatment drops." Participants whose clinical picture worsened or developed a clinical crisis that lead the site clinical team to recommend an out of protocol treatment(s) were classified as "prematurely terminated." Prematurely terminated participants continued treatment within their assigned treatment arm (in so far as clinically possible), as well as all regularly scheduled assessments. Participants who terminated prematurely were distinguished from "study drops" who were participants who refused study treatment and assessments. Stated differently, study drops were defined as those participants who withdrew consent for continued participation in the study.

### Sample Size and Power Estimates

The primary measure used for sample size estimation was the IE's rating of Phase I treatment response. Using chi-square, power estimates for detecting differences in treatment response among the four treatment conditions were computed using the following assumptions: (1) H_a_: P_(SRT) _= 0.60, P_(CBT) _= 0.60, P_(COMB) _= 0.80, and P_(PBO) _= 0.30; (2) sample sizes of 136 for each active treatment condition and 70 for the PBO condition; (3) no adjustment for multiple comparisons; (4) power set at 80%; and (5) alpha = 0.05, two-tailed test. Given these assumptions, power analysis revealed that CAMS was sufficiently powered to detect a 0.19 difference in Phase I response rates between PBO and each active treatment condition and a 0.17 difference in Phase I response rates between COMB and each active monotherapy condition.

### Sampling Frame and Participant Recruitment

CAMS recruited a volunteer sample of children and adolescents between the ages of 7 and 17 years. Inclusion and exclusion criteria are presented in Tables 1 and 2. A complete description of the clinical characteristics of the sample can be found in Kendall and colleagues [43].

**Table 1:** List of Inclusion Criteria

Inclusion Criteria	Rationale
Ages 7 to 17 years inclusive	Matches developmental sensitivity of treatments and study measures
DSM-IV diagnoses of SAD, SoP, or GAD	Disorders of interest
ADIS CSR ≥ 4 for either SAD, SoP, or GAD	Indicates symptom severity/impairment sufficient for DSM-IV diagnosis
IQ estimate > 80	Low IQ may limit the child's ability to profit from CBT
Free from anti-anxiety medications prior to baseline evaluations	Potential confound with study treatments
Outpatient	Inpatient populations are different from sample of interest

**Table 2:** List of Exclusion Criteria

Exclusion Criteria	Rationale
The following Axis I disorders:	These disorders require treatments not provided within the context of the CAMS trial
• Major Depressive Disorder	
• Bipolar Disorder	
• Psychotic Disorder	
• Pervasive Developmental Disorder	
• Uncontrolled ADHD (combined or primarily hyperactive type)	
• Eating Disorders	
• Substance Use Disorders	

Any Axis I disorder (excluding those mentioned above), with an ADIS CSR ≥ to the CSR of the disorders of interest (SAD, GAD, SoP)	Disorders of interest (SAD, SoP, GAD) must be the most severe and disabling conditions affecting the child
School refusal behavior characterized by missing > 25% of school days in most recent term	May require additional or different treatments
Suicidal or homicidal	Unethical to randomize to PBO
Two previous failed trials of an SSRI or a failed trial of an adequate course of CBT for the disorders of interest	Not likely to respond to study treatments; may require additional treatments
Intolerance to sertraline	Risk of side effects/adverse events
Confounding medical condition	Potential medical risk or confounding issue
Pregnancy	Potential risk of medication effects to fetus
Child or adolescent does not speak English	Cannot complete study assessments and CBT

With the exceptions noted below and in Table 2, CAMS investigators sought to enroll a sample of anxious youth representative of the full range of ethnic/minority backgrounds and as similar as possible to those seen in general clinical/hospital practice and community clinical settings. Youth with a co-primary diagnosis (defined as an ADIS CSR equal to that of at least one of the target disorders) for which a different disorder-specific treatment was indicated were not included (i.e., substance abuse disorder, eating disorder). However, to enhance the generalizability of the results, youth with an Axis I disorder(s) with an ADIS CSR less than that of one of the target disorders, with the exception of those disorders listed in Table 2, were included to ensure a broadly representative sample of anxious youth. Given that children with major depressive disorder (MDD) respond to SSRIs and that standard CBT for anxiety disorders does not specifically target symptoms of depression, participants who met DSM-IV criteria for MDD (at any ADIS CSR level) were excluded. This decision was made to ensure a sample whose outcomes could be most clearly interpreted as related to the anxiety disorders of interest.

Using similar procedures, sites recruited participants from mental health pediatric and primary care clinics, community mental health centers, schools, churches, community organizations, and paid and unpaid advertisements in all forms of local media. Special outreach efforts dedicated to enhance minority enrollment were made. These outreach efforts were planned and implemented at each site in consultation with local academic experts and minority community leaders, including educators and clergy. Specific outreach activities included educational talks to schools, churches, and other community groups in minority neighborhoods, articles and paid advertisements in minority-targeted press and media, and direct mail.

English-fluency was a requirement for child enrollment in CAMS, and parents were required to speak sufficient English to provide informed consent for study participation and completion of study treatment and assessment requirements. However, CAMS sites in areas with high percentage of Spanish-speaking families employed bilingual screeners and clinical staff in order to increase the comfort level of bilingual parents and enhance recruitment and retention of these families. In addition, efforts were made at all sites to employ clinical and research staff representative of the ethnic/minority makeup of the local population.

Although, the racial/ethnic diversity of the CAMS sample (21%) is comparable with other published child anxiety treatment studies,[18,19,44] the recruitment of ethnic minority populations into clinical trials remains one of the most significant challenges common to all studies. Establishing effective relationships with the leadership of minority organizations that serve ethnic minority communities can facilitate minority recruitment efforts [45]. Anecdotally, with respect to ethnic minority recruitment efforts in CAMS, challenges faced by investigators were primarily logistical barriers. Most minority participants, for example, had to travel a great distance to participate in the trial. Study reimbursement (paying) for transportation did not seem to enhance enrollment and retention for these participants, suggesting that time was the primary barrier. For future studies, one potential solution to minimize this problem would be to set-up satellite treatment and assessment clinics within local minority communities. Although this solution would likely lead to higher rates of minority participation, it would likely be costly. Further attention to these and other strategies that would enhance minority and ethnic enrollment and engagement is warranted, not only to ensure that research samples are diverse and generalizable, but also because these same barriers that impact study participation likely impact the access and utilization of clinical care in these communities.

### Study Treatments

CAMS treatments reflected current state-of-the-art interventions. Although study protocols established the timing and content of each intervention, treating clinicians were able to work collaboratively with participants and their families to maximize adherence and benefit, and minimize adverse events.

#### Pharmacotherapy

The CAMS medication management strategy was designed to maximize treatment adherence and study participation, enhance and maintain the doctor-patient relationship, instill hope for improvement, and acquire data necessary for medical decision-making without implementing CBT. Medication visits lasted approximately 30 minutes (with the exception of the first which lasted approximately 60 minutes) and were devoted to a review of the participant's symptomatology, overall functioning, response to treatment, and presence of adverse events, all in a context of supportive clinical care.

Pharmacotherapy (PT) visits were scheduled at weeks 1-4, 6, 8, 10, 12 during Phase I. Interim phone visits were scheduled at weeks 5, 7, 9, and 11. Monthly maintenance visits for treatment responders occurred during the six-month follow-up period of Phase II. Consistent with good medical practice, every effort was made to use the most effective and tolerated dose of SRT. Medication was administered daily using a "fixed-flexible" dosing strategy that was linked to the PT therapist-assigned, 7-point CGI-Severity score and the ascertainment of clinically significant side effects. In general, participant's medication dose was adjusted upward in 50 mg/day increments if the clinician-rated anxiety severity on the CGI-S was 3 (mild) or greater. The dose was held, or adjusted downward, if the participant had few anxiety symptoms (CGI-S of 1 or 2) or if there were impairing side effects.

#### Cognitive Behavioral Therapy

CAMS adapted the evidence-based "Coping Cat" CBT protocol [25,46]. Guidelines assisted the therapist in adapting the manual flexibly and in a standardized manner for a client's age and developmental level. "The C.A.T. Project,[47] a version of the Coping Cat modified for use with adolescent participants, allowed therapists to provide developmentally appropriate CBT across the full age range of the study. Across both child and adolescent CBT protocols, the number of session was reduced from 16-20 60-minute treatment sessions (in the original protocols) to 14. Twelve of these sessions were individual child/adolescent sessions and 2 were parent sessions, which were scheduled immediately after the child session at weeks 3 and 5. CBT responders received monthly CBT maintenance sessions during the six-month follow-up period of Phase II.

The first six CBT sessions taught new skills to the child/adolescent (e.g., the FEAR plan), whereas the second six sessions provide opportunities to practice newly learned skills (exposure tasks) within and outside of the sessions. The overall goal of CBT was to teach youth to recognize the signs of unwanted anxiety, let these signs serve as cues for the use of more effective anxiety management strategies, and face rather than avoid anxiety provoking situations.

#### Combination Treatment

Participants in the combination treatment condition (COMB) received all the components from the medication-only and CBT-only treatment conditions, with the exception that the participant, parent(s), and clinician were aware that the child/adolescent was receiving active SRT and active CBT. Pharmacotherapy and CBT visits typically took place on the same day, with the participant seeing the PT therapist first. Clinicians were encouraged to discuss the clinical status of each COMB patient to allow for treatment integration. For example, the PT therapist could increase the dose of SRT (or not), depending on whether the participant was making sufficient progress in CBT. With the exception of one site, COMB treatment visits were held at the same location.

### Patient Safety and Adjunctive Services to Prevent Study Attrition

Participant safety was a foremost consideration, and from a public health point of view, the ascertainment of adverse events in each treatment condition was a critical aspect of the trial. Primary concerns included possible untoward reactions to study treatments and the risk that the participant may not improve or may deteriorate during treatment. CAMS protocols for monitoring safety and providing additional treatment visits to manage clinical crises and concerns that inevitably arise during the course of a trial facilitated standardized, yet flexible, clinically appropriate "best practice" standards and maximized participant retention.

Side effects and adverse events were assessed immediately before each treatment visit by the study coordinator by asking both the child and parent if they had experienced or noticed any health or other problems since the last treatment visit. Responses were recorded and then provided to the treating clinician who reviewed the list with the child and parent to determine its severity, association with study treatments, and actions to be taken by the study team. This 2-stage strategy was used to ensure standardized ascertainment of adverse events across the four treatment conditions.

In response to FDA black box warning regarding the risk for suicidality events associated with SSRIs,[38] and in consultation with NIMH and the CAMS Data Safety and Monitoring Board, a harm to self and others questionnaire was developed and implemented. The participant's treating clinician administered this form at each treatment session to document the onset or change in harm-related ideation or behavior.

To ensure cross-site uniformity in the management of clinically emergent situations, CAMS followed procedures implemented in other pediatric comparative trials [39,40]. Up to 2 additional treatment sessions ("ASAP sessions") were permitted per participant in both Phase I and II to manage any newly emergent clinical needs and facilitate participant retention. Participants whose clinical needs required more than two ASAP sessions per study Phase were "prematurely terminated" by the site team and referred for additional treatment outside the study.

### Assessments

The CAMS assessment battery evaluated the impact of treatment on the presence and degree of anxiety symptomatology, associated comorbid symptoms, and psychosocial functioning across multiple functional domains. Additional assessments included a wide range of demographic variables, comorbid symptomatology, parental psychopathology, family functioning and environment, treatment adherence, cognitive self-talk, and treatment-related expectancies and beliefs. Finally, measures were included for quality assurance purposes and to assess the adequacy of the blind. A summary of primary and secondary assessment measures, and the domains measured, are provided in Table 3.

**Table 3:** Primary and Main Secondary Assessment Measures

Measure	Abbreviation	Domain
**Independent Evaluator**		
Anxiety Disorders Interview Schedule	ADIS-RLV	Diagnostic interview
Clinical Global Impression Scales	CGI-S/I	Severity and improvement
Family History Score Sheet	FAMH1	Family history
Global Assessment Scale for Children	CGAS	Impairment and functioning
Guess Treatment	GUESE	IE blindness
Pediatric Anxiety Rating Scale	PARS	Anxiety severity
Supplemental Services Intake	SSIN	Extra-study interventions
Wechsler Intelligence Scale for Children - III	WISC-III	Intellectual functioning
**Child and parent report: about child**		
Ambiguous Situations Questionnaire	ASQ	Threat bias
Child Anxiety Impact Scale	CAIS	Anxiety related interference
Coping Questionnaire	CQ	Perceived coping ability
Goal Attainment Scale	GAS	Anxiety symptom improvement
Mood and Feelings Questionnaire	MFQ	Depressive symptoms
Multidimensional Anxiety Scale for Children	MASC	Anxiety symptoms
Screen for Child Anxiety Related Emotional Disorders	SCARED	Anxiety symptoms
**Child report only: about self**		
Negative Affectivity Self-Statement Questionnaire	NASSQ	Negative cognition (self-statements/self-talk)
Physical Symptoms Checklist	PSC	Physical symptoms
**Parent report: parent about child**		
Child Behavior Checklist	CBCL	Behavioral problems/social and academic competence
Child and Adolescent Health Screening Report	MEDHXC	Medical history
Peterson Pubertal Developmental Scale	PDS	Pubertal status
Family Burden Assessment Scale	BAS	Burden on child on family
**Parent report: parent about self**		
Brief Symptom Inventory	BSI	Parental psychopathology
State-Trait Anxiety Inventory - A Trait Scale	STAI-T	Parental anxiety
Brief Family Assessment Measure - III	BFAM-III	Family functioning
**Child/Parent report: about treatment**		
Perception of Therapeutic Relationship	PTR	Therapeutic relationship
Satisfaction Questionnaire	SQ	Satisfaction with treatment
**Clinician Measures**		
Adverse Events	AEF	Side effects
Harm to Self and Others	HARM	Suicidal ideation and risks
Session Summary Sheets	SSS	PT and CBT session summary
**Miscellaneous**		
Concomitant Medication/Treatment Log	CML/CTL	Concomitant medication/treatment
Female Menstrual Cycle	FMC	Menstrual status/sexual activity
General Information Sheet	GENINFO	Demographic information
Pre-treatment Expectancy	PTX	Therapist/patient expectancies
Supplemental Services Follow-up	SSFU	Extra-study interventions
Treatment Assignment Reaction	TAR	Treatment expectancies
Vital Signs	VITAL	Vital signs

CAMS participants completed three "full" IE assessment sessions: baseline, week 12, and week 36; and three "partial" IE assessment sessions: weeks 4, 8, and 24. Participants were reimbursed for time and expense involved in completing the assessments in accordance with local IRB regulations.

### Quality Assurance

All study personnel passed their local institutions' required certifications for the ethical conduct of research and HIPAA training. Candidates wishing to be study clinicians (i.e., CBT therapists, PT therapists) and independent evaluators (i.e., IEs) underwent a rigorous certification process which included: (1) a review of credentials (MA or PhD for CBT therapists; MD or NP for PT therapists; and MA, RN, PhD, or MD for IEs) and clinical experience treating anxious youth; (2) reading study related materials; (3) passing a test on the treatment and study protocols (passing was defined as a score of 80% or greater); (4) completing a training workshop; and (5) passing a videotape or audiotape review of a training case(s) for evaluation of fidelity and competence by the QA reviewers (i.e., as noted earlier, Temple University conducted QA for CBT sessions, UCLA for PT sessions, and NYSPI for IE assessments). After certification, CBT, PT, and IE staff received onsite supervision by a study supervisor and ongoing cross-site supervision during separate and independent one-hour conference calls. CBT therapists had weekly onsite and cross-site supervision, IEs had weekly onsite supervision and every other week cross-site supervision, and PT therapists had monthly onsite supervision and every other week cross-site supervision. In addition to the supervision provided onsite and cross-site via conference call, there was an initial in person start-up training workshop (3 days for CBT and IEs) followed by annual in-person recalibration and training sessions for all study staff throughout the 6-years of the trial. These procedures allowed the investigative team to correct any drift and address the management of clinical issues and situations in a similar fashion that could potentially impact the integrity of the trial while facilitating a collaborative study culture across sites.

### Design Weaknesses and Challenges

The primary weakness of the CAMS design, and other clinical trials similar to CAMS (e.g., MTA, TADS, and POTS), is that the CBT and COMB participants were not blinded. The only double-blinded treatment conditions were SRT and PBO. CBT participants knew they were receiving CBT and COMB participants knew they were receiving both CBT and SRT. This leaves results from the CAMS trial open to the criticism that participant and family expectancy effects may potentially bias outcomes. However, as argued in other similar comparative treatment trials,[48] the decision not to blind certain treatment conditions are design choices rather than necessarily design flaws. In the case of CAMS, the design chosen represented the best balance between scientific rigor, potential for public health impact, ecological validity, feasibility of implementation, and cost. Moreover, CAMS investigators recognized this limitation in the design chosen and took deliberate steps to minimize threats to the internal validity of the trial by using IEs who were blind to participant's treatment assignment to measure outcomes.

The CAMS study experienced challenges, as well as successes, when it came to monitoring adverse events. The CAMS Data Safety Monitoring Board (DSMB) initially approved an adverse event monitoring policy based on procedures that were used in the TADS study [40]. This policy consisted of only recording adverse events that caused moderate to severe distress or resulted in functional impairment. The implication of the policy was that mild adverse events that did not result in functional impairment were not recorded. However, several months into the trial the DSMB reversed its decision and mandated the collection and reporting of all adverse events, including mild events. Further complicating adverse event collection procedures, several years into the study the DSMB required CAMS clinicians to systematically query and collect information about the presence of any harm related adverse events from all study participants and parents at each study visit (i.e., non-suicidal self-harm, suicidal ideation, homicidal ideation, suicidal behavior, and homicidal behavior). Although moderate and severe adverse event data were systematically collected throughout the entire trial, the shifting methods used to elicit and record adverse events complicates the conclusions that can be made about the presence or absence of adverse events throughout the entire trial for all subjects.

Despite this limitation, CAMS utilized an adverse event procedure that was very effective. In response to the finding in the TADS study that CBT therapists did not monitor adverse events as closely as did the PT therapists, a different procedure was implemented in CAMS for eliciting adverse events than is typically used in clinical trials. In most clinical trials, clinicians (whether a psychologist or psychiatrist) asks the participant or parent(s) if they noticed any changes since their last treatment visit. However, to avoid the problem found in TADS, the elicitation of adverse events was assessed immediately before each treatment visit by the site project coordinator across all treatment conditions. This ensured that the elicitation of adverse events did not vary by treatment condition. This novel adverse event monitoring procedure was effective in standardizing the elicitation of adverse events across different treatment conditions and will allow for comparisons of relative safety across the four treatment conditions.

## Conclusion

Following the MTA, POTS, TADS and TORDIA[27,39,40,49]--all randomized controlled trials in which most CAMS investigators participated--CAMS is now the fifth large-scale multicenter comparative pediatric treatment trial funded by the NIMH in the last decade. CAMS benefitted from lessons learned in these earlier studies and as a result used best available methods in experimental design, trial execution and data analysis. With respect to its public health goals, CAMS directly addresses the lack of research examining the efficacy of CBT alone and PT alone in the same population of anxious children that were recruited, assessed, and treated using similar methodologies. CAMS also addressed the critical question of whether combining treatments provides additional benefit. Results of the acute outcomes of the CAMS trial showed that all active treatments were superior to pill PBO, that combination treatment was superior to the monotherapies, and that the monotherapies were equivalent. These findings are encouraging and suggest that there are three effective interventions for the treatment of three of the most common anxiety disorders in child and adolescent populations. The CAMS assessment protocol allowed for the evaluation of changes in the symptom profile, functional outcomes, and adverse events, as well as putative mediators and moderators of response, in an explicit multi-measure, multi-informant fashion and will provide a platform to address many public health questions. For example, results from the acute outcomes reveals that that suicidal events are far less common in anxious youth than in patients with depression,[50] with no additional sertraline-associated suicidal events reported [15]. Although the CAMS trial used experienced and well-trained and closely monitored clinicians, the CAMS manual-based protocols were designed to directly translate into community practice making the findings more relevant than they would have been had less readily available treatments been used.

The primary findings from the CAMS suggest both CBT and SRT reduced the severity of anxious symptoms in children and adolescents diagnosed with moderate to severe SAD, GAD or SoP; however, the combination of the two therapies showed the most benefit. Subsequent papers reviewing secondary outcomes, the durability and safety of each treatment and moderators and mediators of study outcome will inform practice-relevant questions regarding the treatment of youth with anxiety disorders.

## List of Abbreviations

ADHD: Attention Deficit Hyperactivity Disorder; ADIS: Anxiety Disorder Interview Schedule; CAMS: Child/Adolescent Anxiety Multimodal Study; CBT: Cognitive Behavioral Therapy; CGI-I: Clinical Global Impression-Improvement; CGI-S: Clinical Global Impression-Severity; COMB: Combination Therapy; DHHS: Department of Health and Human Services; DSM-IV-TR: Diagnostic and Statistical Manual of Mental Disorders-IV Edition-Text Revised; DSMB: Data Safety Monitoring Board; EC: Executive Committee; FDA: Food and Drug Administration; GAD: Generalized Anxiety Disorder; IE: Independent Evaluator; ITT: Intent-To-Treat; MDD: Major Depressive Disorder; NIH: National Institute of Health; NIMH: National Institutes of Mental Health; NYSPI: New York State Psychiatric Institute; OCD: Obsessive-Compulsive Disorder; PARS: Pediatric Anxiety Rating Scale; PBO: Pill Placebo; PT: Pharmacotherapy; QA: Quality Assurance; RCT: Randomized Clinical Trial; SAD: Separation Anxiety Disorder; SC: Steering Committee; SoP: Social Phobia; SRT: Sertraline; SSRI: Selective Serotonin Reuptake Inhibitor; UCLA: University of California at Los Angeles

## Competing interests

In the past two years, all other authors have received grant support from the NIH/NIMH and all authors acknowledge that they received support from Pfizer Inc in the form of free medication and matching placebo for this study. SNC receives consulting fees and grant support from the Tourette Syndrome Association. JTW has received consulting fees from Eli Lilly and JAZZ Pharmaceuticals and lecture fees from CMP Media, Medical Education Reviews, McMahon Group, DiMedix, and the Tourette Syndrome Association. He has received free drug and matching placebo from Lilly, and free drug from Abbott for NIMH-funded clinical trials. He has received fees for consultation with defense counsel and submission of written reports in litigation involving GlaxoSmithKline. AMA receives royalties from Oxford University Press for the Anxiety Disorders Interview Schedule for DSM-IV, Child and Parent Versions (though not for CAMS) and for manuals not used in this study; and royalties from the Guildford Press. JCP has received grant support from the Obsessive Compulsive Foundation, the Eisner Foundation, grant and travel support from the Tourette Syndrome Association, royalties for treatment manuals on childhood obsessive compulsive disorder and tic disorders (from Oxford University Press) and from other books on child mental health (from Guilford Press and APA Books), and speaker honoraria from Janssen-Cilag. BB has received grant support from the Fine Foundation and has participated in forums sponsored by JAZZ Pharmaceuticals, Solvay Pharmaceuticals Inc, and Abcomm Inc. He has given paid talks on the topic of childhood bipolar disorder at a meeting sponsored by Solvay and receives royalties for a book on children with bipolar disorder from Random House, Inc. JTS is a full-time employee of the NIMH/NIH/DHHS. The views expressed in this article are those of the authors and do not necessarily represent the official views of the NIMH, the NIH, or the DHHS. GSG has received additional grant support from the Obsessive Compulsive Foundation. MAR has grant support from Neuropharm, Boehringer Ingelheim Pharmaceuticals, and Wyeth Pharmaceuticals. She is a consultant to Wyeth and receives royalties from APPI for a book chapter on pediatric anxiety disorders. JTM is a paid consultant for Sanofi-Aventis and Wyeth, has received lecture fees from Shire and UCB, and has additional grant support from Aspect, Johnson & Johnson, Bristol-Myers Squibb, and Eli Lilly. BDW has grant support from Baystate Health, Somerset Pharmaceuticals, and GlaxoSmithKline. SI receives fees as a statistical consultant from Stanford University and Westinghouse Corporation. PCK receives royalties from the publication of the anxiety treatment materials (not from this study) and from books on child mental health from Workbook Publishing. JSM is a consultant or scientific advisor to Eli Lilly, Pfizer, Wyeth, Johnson and Johnson, and GlaxoSmithKine. He is a stockholder in MedAvante, the author of the Multidimensional Anxiety Scale for Children (MASC) for which he receives royalties (though not for CAMS), has received study drug for TADS from Eli Lilly, receives research funding from NARSAD and from Pfizer, receives book royalties from Guilford Press and from Oxford University Press, and was a member of a DSMB overseeing research conducted by Astra-Zeneca or Johnson & Johnson.

## Authors' contributions

All listed authors made substantive intellectual contributions to the conception, design, and implementation of the study. In addition, SNC was principle investigator of the data center, secretary on the executive committee, and drafted the manuscript. JTW was principle investigator of the Johns Hopkins site, chair of the executive committee, and helped to draft the manuscript. AMA was principle investigator of the New York State Psychiatric Institute site, co-chair of the executive committee, chair of the independent evaluator subcommittee, oversaw independent evaluator quality assurance ratings, and helped to draft the manuscript. JCP was principle investigator of the University of California at Los Angeles site, chair of the assessment subcommittee, oversaw psychopharmacology quality assurance ratings, and helped to draft the manuscript. BB was principle investigator of the Western Psychiatric Institute and Clinic site and initially set-up and oversaw the data center for several years. JTS was the National Institutes of Mental Health representative on the trial, member of the executive committee, and helped to draft the manuscript. GSG was co-investigator of the Johns Hopkins site and site CBT supervisor. MAR was a co-investigator of the New York State Psychiatric Institute site, chair of the psychopharmacology subcommittee, and site psychopharmacology supervisor. JTM was co-investigator of the University of California at Los Angeles site and chair of the genetics subcommittee. BDW was a co-investigator of the New York State Psychiatric Institute site. SI was chair of the statistics subcommittee. PCK was principle investigator of the Temple University site, chair of the CBT subcommittee, oversaw CBT quality assurance ratings, and helped to draft the manuscript. JSM was principle investigator of the Duke University Medical Center site. All authors read and approved the final manuscript.
